# Teneurins: Mediators of Complex Neural Circuit Assembly in Mammals

**DOI:** 10.3389/fnins.2019.00580

**Published:** 2019-06-05

**Authors:** Catherine A. Leamey, Atomu Sawatari

**Affiliations:** Discipline of Physiology, School of Medical Sciences and Bosch Institute, Faculty of Medicine and Health, The University of Sydney, Sydney, NSW, Australia

**Keywords:** Ten-m/Odz/teneurin, visual pathway, chemoaffinity, development, hippocampus, striatum, neural circuits

## Abstract

The teneurins (Ten-m/Odz) are a family of evolutionarily ancient transmembrane molecules whose complex and multi-faceted roles in the generation of mammalian neural circuits are only beginning to be appreciated. In mammals there are four family members (Ten-m1-4). Initial expression studies in vertebrates revealed intriguing expression patterns in interconnected populations of neurons. These observations, together with biochemical and over-expression studies, led to the hypothesis that homophilic interactions between teneurins on afferent and target cells may help to guide the assembly of neural circuits. This review will focus on insights gained on teneurin function *in vivo* in mammals using mouse knockout models. These studies provide support for the hypothesis that homophilic interactions between teneurin molecules can guide the formation of neural connections with largely consistent results obtained in hippocampal and striatal circuits. Mapping changes obtained in the mouse visual pathway, however, suggest additional roles for these glycoproteins in the formation and specification of circuits which subserve binocular vision.

## Introduction

The idea that groups of afferent and target neurons positioned at locations remote from each other could set up precise, ordered patterns of connectivity due to the affinity of chemicals expressed on or by these cells was postulated formally by Roger Sperry in his chemoaffinity hypothesis (Sperry, 1963). Over the last few decades, a few families of molecules that exhibit expression patterns which fit largely with his predictions have been identified, with notable examples including the Ephs/ephrins, cadherin and immunoglobulin superfamilies (McLaughlin and O’Leary, 2005; Zipursky and Sanes, 2010). One of the more recent entrants to this stage is the teneurins. In a range of different species and brain circuits, these molecules were found to exhibit distributions across afferent and target fields which pointed to the idea that they may indeed help to determine patterns of neural connectivity (e.g., Rubin et al., 1999, 2002; Zhou et al., 2003; Li et al., 2006; Leamey et al., 2008; Carr et al., 2013, 2014; Bibollet-Bahena et al., 2017; Cheung et al., 2019). Over recent years, genetically modified mice have been generated which have enabled these ideas to be tested *in vivo*. The focus of this article is to review what has been learnt from these studies. As will be discussed below, they show key roles for teneurin molecules in regulating the patterns of connectivity in multiple neural circuits, including visual, hippocampal, and striatal networks. Compelling evidence that homophilic interactions between teneurins on axons and targets help to specify precise patterns of connectivity will be described. Evidence that teneurins also play other important roles in mediating appropriate wiring and synaptic efficacy, including interactions with, and regulation of the expression of other molecules will also be presented.

## Topographically Corresponding Gradients Mediate Precise Matching of Neural Connections Via Homophilic Interactions

Teneurins exhibit differential expression patterns within neural circuits. In the chick visual system, for example, Ten-m1 and Ten-m2 were found to be differentially expressed by the tectofugal and thalamofugal pathways, respectively (Rubin et al., 1999, 2002). Dynamic and differential, but partially overlapping, expression patterns have also been observed in the nervous system of zebrafish, with particularly strong expression of Ten-m3 and Ten-m4 (Mieda et al., 1999; Cheung et al., 2019). In this species, expression of Ten-m3 in the amacrine and ganglion cells of the developing retina is important for the formation of intraretinal circuitry (Antinucci et al., 2013, 2016). In addition to different Ten-ms being selectively expressed by specific pathways, topographically corresponding gradients of expression have also been observed at multiple levels within given circuits, suggesting a role in generating precise patterns of connectivity between remotely located afferent and target fields. The most notable examples of this are the expression patterns of Ten-m3 in the developing visual, hippocampal, and striatal circuits in mice.

Initial descriptions of the expression patterns of teneurins in the cortex of the mouse described high levels of Ten-m2, Ten-m3, and Ten-m4 in caudal regions of cortex, with Ten-m1 expressed in more rostral areas (Li et al., 2006; Leamey et al., 2008). Expression in the caudal domain included the primary visual cortex, multiple subregions of the hippocampus and associated cortical areas, as well as intriguing expression patterns in the thalamus and striatum (Zhou et al., 2003; Li et al., 2006; Leamey et al., 2008; Tran et al., 2015; Bibollet-Bahena et al., 2017).

**FIGURE 1:**
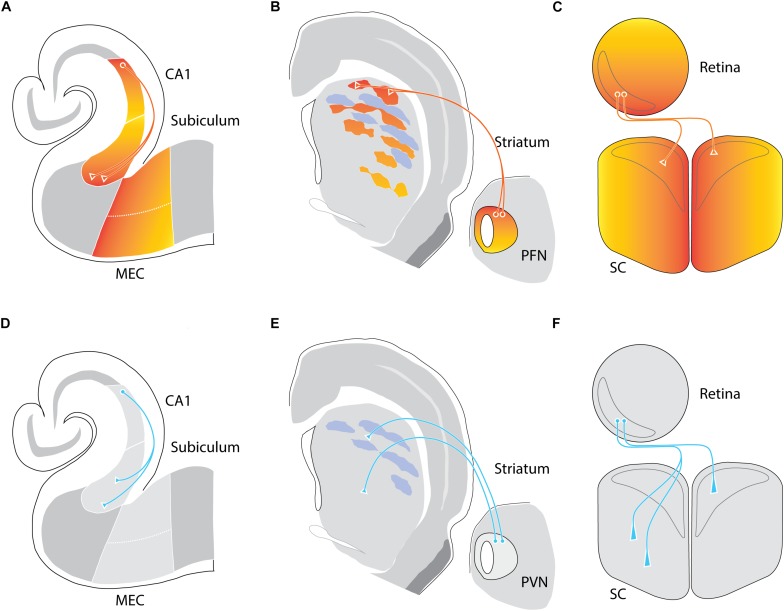
Removal of Ten-m3 leads to connectivity changes consistent with mostly, but not exclusively, a role as a homophilic chemoaffinity molecule. **(A–C)** Ten-m3 is expressed in topographically corresponding gradients in multiple neural circuits. Wild-type (WT) expression of Ten-m3 depicted as colored gradients (red-high, yellow-low) within the hippocampus **(A)**, thalamostriatal **(B)**, and retinocollicular **(C)** pathways of mice. **(A)** Expression is high in proximal (with respect to incoming Schaffer collaterals) CA1, distal (with respect to CA1) subiculum, and proximal (with respect to the subiculum) medial entorhinal cortex (MEC) borders. Connectivity between CA1 and subiculum follows a pattern consistent with homophilic chemoattraction. **(B)** Ten-m3 striatal expression exhibits an overall high dorsal to low ventral gradient, albeit in a patchy manner. A similar high dorsal to low ventral gradient is observed in the parafascicular nucleus (PFN), one of the key sources of thalamic input to the striatum. Here, projections from dorsolateral PFN target Ten-m3-positive patches in dorsolateral striatum. **(C)** Retinal expression of Ten-m3 follows a high ventral, low dorsal gradient. The superior colliculus (SC) in turn expresses the glycoprotein in a high medial to low lateral gradient. Both ipsilateral and contralateral retinal projections from the binocular ventrotemporal crescent (VTC; outlined in gray) target regions within rostromedial binocular SC (also outlined in gray), with contralateral projections tending to terminate in slightly rostral and medial areas compared to ipsilateral termination zones in the opposite hemisphere. **(D–F)** Removal of Ten-m3 leads to axonal miswiring. **(D)** In the hippocampus, deletion of Ten-m3 results in CA1 projections exhibiting a greater spread of termination, targeting distal, as well as more proximal areas within subiculum. **(E)** Dorsolateral PFN projections terminate in more ventral striatal areas in Ten-m3 KOs. In addition, there is a loss of the patchy distribution of thalamostriatal terminals. Both hippocampal, as well as thalamostriatal connectivity exhibit changes consistent with the removal of a homophilic signal. **(F)** Ipsilateral retinal projections targeting the SC are also miswired, with terminals detected in more lateral, as well as posterior locations. Contralateral retinal termination patterns show only subtle alterations with terminal zones narrowed mediolaterally and elongated along the anterior–posterior axis. Thus, for the retinocollicular pathway, the change in wiring can only partially be explained by the removal of a homophilic gradient, suggesting other downstream factors are contributing to proper topographic mapping of this pathway (based on Dharmaratne et al., 2012; Tran et al., 2015; Berns et al., 2018).

While both Ten-m2 and Ten-m4 displayed fairly uniform expression across given subregions of the hippocampus, Ten-m3 showed evidence of differential expression within these areas (Zhou et al., 2003; Li et al., 2006; Leamey et al., 2008). Recent work has confirmed the presence of a gradient of Ten-m3 across three interconnected regions: CA1, subiculum, and entorhinal cortex (Berns et al., 2018). Further, the gradients of Ten-m3 are topographically aligned across these regions: medial entorhinal cortex, proximal CA1, and distal subiculum, all express high levels of Ten-m3 (Figure 1A), and are connected to each other. In contrast, the lateral entorhinal cortex, distal CA1, and proximal subiculum circuit are similarly interconnected and all exhibit low levels of Ten-m3 expression (Berns et al., 2018). Further, this paper showed that multiple other interconnected regions of the hippocampal circuit including the mammillary bodies, anteroventral thalamic nucleus, and pre- as well as para-subiculum also display gradients of Ten-m3 (Bibollet-Bahena et al., 2017; Berns et al., 2018).

A topographic correspondence in the expression patterns of Ten-m3 between connected areas has also been found for the thalamostriatal pathway (Tran et al., 2015). In the striatum, Ten-m3 expression is patchy but distributed in an overall high dorsal to low ventral gradient within the matrix. A topographically corresponding high-dorsal to low-ventral gradient of Ten-m3 expression pattern is found in the parafascicular thalamic nucleus, a major source of input to the striatal matrix (Figure 1B). Interestingly, thalamostriatal terminals have a patchy distribution that overlaps with Ten-m3-positive regions (Tran et al., 2015).

**FIGURE 2:**
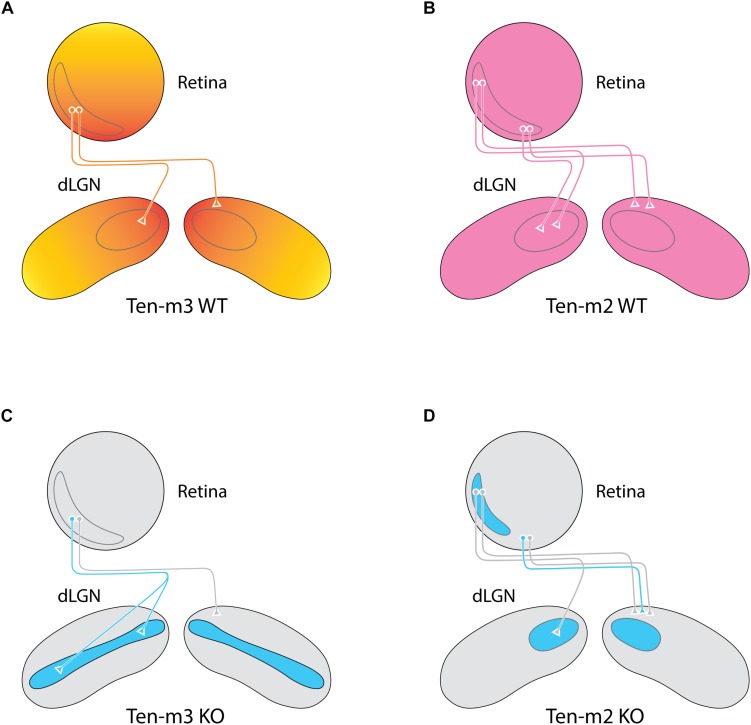
Deletion of Ten-m3 and Ten-m2 lead to specific, complementary wiring deficits within the retinogeniculate pathway. **(A,B)** Ten-m3 exhibits a high dorsomedial to a low ventrolateral gradient in coronal sections of the dLGN complimenting the high ventral, low dorsal expression gradient present in the retina **(A)**. In contrast, Ten-m2 exhibits a uniform expression pattern across both the retina and dLGN **(B)**. Binocular retinal projections from the VTC (gray outline) target complimentary, topographically aligned regions within the dLGN of both Ten-m2 and Ten-m3 WTs. **(C)** Ten-m3 deletion results in a dramatic miswiring of ipsilateral retinal projections, with aberrant terminals targeting the normally monocular ventrolateral region of the nucleus. Contralateral projections remain largely unaltered, leading to a disruption of the topographical overlap between the inputs from both eyes. **(D)** Removal of Ten-m2, in contrast, results in ipsilateral projections from the ventral portion of the VTC taking on a contralateral fate, effectively reducing the size of both the VTC and the ipsilateral recipient area within the dLGN. Thus, Ten-m2 is required for appropriate guidance at the optic chiasm, whereas Ten-m3 is required for appropriate topographic mapping with target structures (based on Leamey et al., 2007; Young et al., 2013).

The observation of a high caudal to low rostral expression gradient across the visual cortex (Leamey et al., 2008) sparked an investigation of other areas within this sensory pathway. The presence of a high ventral to low dorsal gradient of Ten-m3 across the retina has been revealed, including in retinal ganglion cells (RGCs), which provide output to central visual structures (Leamey et al., 2007). Even more compellingly, the two main topographically organized primary targets of RGC axons, the dorsal lateral geniculate nucleus (dLGN) and superior colliculus (SC), also exhibit graded expression patterns that are high in the areas which received input from ventral retina (dorsal dLGN and medial SC, respectively) and low in regions that are driven by dorsal retina (ventral dLGN and lateral SC) (Leamey et al., 2007; Dharmaratne et al., 2012; Figures 1C, 2A). Interestingly, similar gradients were found in the marsupial wallaby, suggesting a conservation of Ten-m3 function across mammalian species widely separated by evolution (Carr et al., 2013, 2014).

The remarkable consistency in these patterns within a range of neural circuits across Mammalia pointed strongly to the idea that Ten-m3 may function as a classic chemoaffinity molecule, promoting connectivity between afferent axons and target cells with corresponding levels of expression. While *in vitro* studies have provided general support for this idea, showing that teneurins promote cellular adhesion *in vitro* (Rubin et al., 2002; Berns et al., 2018), proof of a role in mediating connectivity requires *in vivo* manipulations.

Analysis of the Ten-m3 knockout mouse, generated by deletion of exon 4 (Leamey et al., 2007), has demonstrated functionally important roles for this molecule in the formation of appropriate connectivity. Global Ten-m3 removal results in a loss of precision in thalamostriatal connectivity (Figure 1E). Consistent with the pattern of Ten-m3 expression, deletion of the active gene results in changes in the overall topography of the pathway, as well as inducing the normally tight clusters of thalamostriatal terminals observed in wild types (WTs) to become more diffuse (Tran et al., 2015). Subtle changes in the accuracy of contralateral retinocollicular projections have also been observed, with terminal zones exhibiting a narrowing across the mediolateral, as well as an elongation along the rostrocaudal axes of the SC (Dharmaratne et al., 2012; Figure 1F). In the hippocampus, the targeting of proximal CA1 to distal subiculum is less precise in global Ten-m3 KOs than in WTs (Berns et al., 2018; Figure 1D). This work provides good evidence that Ten-m3 acts homophilically to promote accurate connectivity between areas that express similar levels of the protein in a variety of circuits, consistent with the notion that it serves as a classic chemoaffinity molecule.

Several questions remain unanswered, however. Most notably, is Ten-m3 expression required in afferent axons, target cells, or both? The recent development of a conditional Ten-m3 KO mouse has enabled this issue to be elegantly addressed in the hippocampal circuit. Deletion of Ten-m3 in either the afferent or the target cells alone is sufficient to disrupt the usual pattern of connectivity between CA1 and subiculum (Berns et al., 2018) and reinforces the suggestion that homophilic interactions are important for Ten-m3 function. Of interest, the phenotype observed when Ten-m3 is deleted only from a subregion of subiculum suggests that Ten-m3-positive axons will avoid terminating in areas they would normally innervate to target Ten-m3-positive targets. Curiously, while there are multiple, differentially spliced variants of Ten-m3 expressed in the circuit, all except one is able to mediate homophilic interactions between cells, as well as cellular aggregation (Berns et al., 2018). Interestingly, this exception was still able to promote adhesion with cells that express latrophilin-3, a molecule that acts by forming heterophilic bonds with Teneurins across synapses (Boucard et al., 2014; Sando et al., 2019).

## Beyond Homophilic Adhesion: Teneurins in the Formation of Binocular Visual Circuits

The studies described above provide compelling evidence that the graded expression pattern of Ten-m3 is fundamentally important in promoting precise patterns of connectivity within neural circuits, supporting its role as a homophilic adhesion molecule. Evidence suggests, however, that this may be only one component of Ten-m3’s function, at least for the formation of binocular visual circuits.

As noted above, topographically connected regions of the early visual pathway show similar expression levels of Ten-m3. Evidence of altered mapping is apparent in the contralateral retinocollicular pathway of Ten-m3 KOs (Dharmaratne et al., 2012). It should be pointed out, however, that these changes are quite subtle when compared to the much more dramatic miswiring observed in the mapping of both the ipsilateral retinocollicular (Dharmaratne et al., 2012) and retinogeniculate pathways in these mice (Leamey et al., 2007). The ipsilaterally projecting RGC population originates from a subset of cells in the peripheral ventrotemporal crescent (VTC) of the retina (Drager and Olsen, 1980), and usually projects exclusively to a patch in the dorsomedial region of the thalamic nucleus (Figure 2A). In Ten-m3 KO mice, it has been found that while the ipsilateral pathway originates from the same region of the retina as in WTs, its terminals form an elongated strip that extend from dorsomedial to far ventrolateral dLGN (Leamey et al., 2007; Figure 2C). Ipsilateral retinocollicular projections in KOs also exhibit highly aberrant wiring, with terminals normally confined to rostral and medial areas targeting more caudolateral locations (Dharmaratne et al., 2012; Figure 1F). The degree of change observed indicates a profound difference in the effect of Ten-m3 on the targeting of ipsilateral and contralateral retinal projections.

Since the ipsilateral projection arises from, and projects to, regions associated with high levels of Ten-m3 expression, the changes observed in KOs are broadly consistent with the idea that this molecule promotes the formation of synaptic contacts between areas with similar expression levels, and thus helps to set up topographical alignment within the visual pathway. A critical look, however, suggests a more complex role. Notably, the expression of Ten-m3 has a broad ventrodorsal retinal gradient, but shows no difference between temporal and nasal regions (Dharmaratne et al., 2012). Further, Ten-m3 expression is clearly not restricted to the ipsilateral population, which comprises only a fraction of the RGCs within the VTC (Drager and Olsen, 1980). The much more pronounced effect of Ten-m3 deletion on ipsilateral mapping is therefore not well-correlated with its expression pattern. Further, although a loss of precision in the pattern of connectivity of ipsilateral projections is observed along the axis of Ten-m3 expression, much more pronounced changes are apparent along the axis that is orthogonal to the Ten-m3 gradient (Dharmaratne et al., 2012). These observations suggest that Ten-m3 may have additional mechanisms of action in the targeting of ipsilateral projections.

An investigation into the development of the retinogeniculate projection revealed that the mistargeting of ipsilateral RGC terminals in the dLGN in Ten-m3 KOs is preceded by an abnormally early exit of retinal axons from the optic tract into the nucleus (Glendining et al., 2017). In WT mice, ipsilateral axons remain largely confined to the optic tract until they reach the dorsal half of the dLGN. In Ten-m3 KO mice, however, the ipsilateral axons leave the optic tract to enter the nucleus near its ventral border. This change is difficult to explain as a direct consequence of the deletion of an attractive adhesion molecule expressed in the dorsal part of the dLGN. Even the demonstration that cleavage products of teneurins can form soluble proteins that impact axon guidance (Vysokov et al., 2018) does not really help here, as the attractive molecule has been deleted, yet the axons enter a region from which they would usually be repelled. The avoidance of ventrolateral dLGN by ipsilateral retinal axons has been shown to involve repellent interactions between EphA receptors and their ligands (Pfeiffenberger et al., 2005), which are also expressed in gradients in the retina, SC, and dLGN (Feldheim et al., 1998, 2000; Frisen et al., 1998). Intriguingly, the expression gradients of the EphA/ephrinA families are orthogonal to the Ten-m3 gradient, so an interaction with this pathway would help to explain both the axis and direction of change observed in the KO phenotype. While no changes in expression levels of most of the relevant EphA/eprhinA family members were detected, a significant reduction in the expression of the EphA7 receptor has been revealed in Ten-m3 KO mice on the day of birth (Glendining et al., 2017) which may help to account for the observed changes in ipsilateral termination. *In vitro* studies have shown that the intracellular domain of Ten-m2 may be cleaved and translocate to the nucleus where it interacts with transcription factors such as Zic1 (Bagutti et al., 2003). Since the intracellular domain of Ten-m3 contains both a potential cleavage site and a nuclear localization signal (Tucker et al., 2012; Leamey and Sawatari, 2014), interactions with transcription factors seem likely. Indeed, a pull-down assay has demonstrated that the intracellular domain of Ten-m3 interacts with Zic2 (Glendining et al., 2017), a transcription factor which is a key determinant of ipsilateral identity (Herrera et al., 2003; Garcia-Frigola et al., 2008; Lee et al., 2008). Moreover, mRNA for both Zic2, and its downstream mediator of ipsilateral axonal guidance at the chiasm, EphB1 (Williams et al., 2003) are upregulated in Ten-m3 KOs (Glendining et al., 2017). Thus, a key component of the role of Ten-m3 in the formation of binocular visual circuits is likely to arise via the interaction with, and regulation of, other signaling molecules.

Interestingly, since Zic2 and EphB1 promote ipsilateral identity and retinal axon guidance, respectively (Herrera et al., 2003; Williams et al., 2003; Lee et al., 2008), the upregulation of these molecules seen in Ten-m3 KOs at around the day of birth (Glendining et al., 2017) could be expected to result in an increase in the size of the ipsilateral projection in Ten-m3 KOs. Retrograde tracing showed, however, no change in either the number or distribution of ipsilaterally projecting RGCs in adult mice lacking Ten-m3 (Leamey et al., 2007). It is possible that the upregulation of Zic2 in Ten-m3 KOs occurs too late to induce a change in the laterality of projections, although since Zic2 expression peaks at embryonic day 16.5 (Herrera et al., 2003) and Ten-m3 is usually expressed at high levels in the retina by this time (Dharmaratne et al., 2012), this possibility seems unlikely. Alternatively, rather than more cells expressing Zic2 and EphB1, the level of expression of these molecules within individual RGCs may be increased. If this was the case, given the known role EphB molecules in retinal mapping (Hindges et al., 2002; McLaughlin et al., 2003), an increase in EphB1 would be expected to cause ipsilaterally projecting retinal axons to map more laterally in the SC in Ten-m3 KOs than in WTs. Interestingly, this fits with what has been observed (Figure 1F; Dharmaratne et al., 2012).

As noted above, two other members of the Teneurin family, Ten-m2 and Ten-m4, are also highly expressed in the mouse visual cortex at around the time of birth (Li et al., 2006; Leamey et al., 2008). Analysis of Ten-m2 KOs has revealed a key role for this family member in the formation of binocular visual circuits which complements that of Ten-m3. Similar to what is observed in the hippocampal circuit, Ten-m2 does not display an obvious differential expression within the visual pathway that would suggest a role in topography. Rather, the molecule appears to be uniformly distributed across the RGC layer and within the SC, dLGN, and V1 in perinatal mice (Young et al., 2013). Despite this fairly uniform distribution pattern within the visual pathway (Figure 2B), analyses of the Ten-m2 KO yielded evidence of a highly specific defect which again impacted the formation of the binocular visual circuit. In Ten-m2 KO mice, the ipsilateral visual pathway is found to be reduced in size. Further, the loss of ipsilaterally projecting RGCs is only observed in the ventral part of the VTC (Young et al., 2013; Figure 2D). This highly specific role of Ten-m2, which does not correlate easily with its expression pattern, suggests that, as with Ten-m3, its role in the formation of binocular visual circuits is likely to involve interactions with other molecules. Given its association with the ipsilateral pathway, potential changes in Zic2 and EphB1 have been investigated. While no difference in Zic2 is observed, a down-regulation of EphB1 is seen specifically in the ventral part of the VTC, suggesting that Ten-m2 may work downstream of, or in parallel with, Zic2.

## Functional Impacts From Loss of Teneurin Function

The impact of the loss of teneurin function on behavior in KO mice has been best characterized for the visual pathway. In Ten-m3 KOs, the mistargeting of ipsilateral retinal axons is associated with profound visual deficits. Assessment of behavior that requires patterned vision reveals performance at chance levels, although the mice show an ability to distinguish between dark and light (Leamey et al., 2007). Interestingly, acute inactivation of neural activity in one eye significantly improves performance on tasks requiring patterned vision. This suggested that the misalignment of ipsilateral and contralateral visual inputs to one hemisphere may lead to a suppression of activity in V1. A subsequent investigation supported this by demonstrating that binocular, but not monocular, drive to V1 is significantly reduced in Ten-m3 KOs (Merlin et al., 2013). Similar mechanisms may, at least in part, contribute to the visual disorders associated with Ten-m3 mutations in humans (Aldahmesh et al., 2012). Moreover, the Ten-m3 KO is not the only teneurin model that exhibits defects of visual function. The loss of ipsilateral projections from the ventral part of the VTC in Ten-m2 KOs is also associated with a reduced ability to discriminate visual stimuli presented to dorsal visual field (Young et al., 2013).

While the Ten-ms clearly play an important role in the formation of binocular visual circuits in mice, it should be pointed out that they are also expressed in the visual pathway of zebrafish and chicks (Rubin et al., 1999, 2002; Antinucci et al., 2013; Cheung et al., 2019), species which have little or no ipsilateral retinal projection. In zebrafish, which have entirely crossed retinal projections and lack binocular overlap, Ten-m3 has been shown to contribute to the specification of RGC connectivity and function (Antinucci et al., 2013, 2016), consistent with its role as a homophilic adhesion molecule. The manner in which Ten-ms help to regulate the formation of binocular circuits of mice may be an evolutionary “add-on”, critical to the alignment and function binocular visual circuits in mammalian species. More information regarding the expression of Ten-ms in mammals with varying degrees of binocularity would be helpful as a first step in assessing this possibility.

The impaired thalamostriatal targeting in Ten-m3 KOs is also associated with functional changes. Notably, while there is no difference in initial and post-acquisition performance levels of a simple motor task, the rate of learning is negatively affected in Ten-m3 KOs (Tran et al., 2015). Although changes in spatial learning might also be expected following the miswiring in hippocampal connectivity (Berns et al., 2018), this has yet to be reported in Ten-m3 KOs.

Ten-m1 is highly expressed in the olfactory bulb and cortex (Allen Brain Atlas). Deletion of Ten-m1 has been shown to affect the KOs ability to detect appetitive and aversive odors (Alkelai et al., 2016). Although less well-characterized in mice compared to the other Ten-ms, this finding correlates with the identification of Ten-m1 in patients with congenital anosmia, as well as an important role for teneurins in the establishment of olfactory circuits in *Drosophila* (Hong et al., 2012).

While a thorough behavioral characterization has yet to be conducted on Ten-m4 KOs, this teneurin has been linked to bipolar disorder and schizophrenia in humans (Heinrich et al., 2013; Ivorra et al., 2014). The insertion of a transgene which disrupts Ten-m4 expression in mice has been shown to impede oligodendrocyte differentiation. This is associated with reduced myelination and tremors (Suzuki et al., 2012). Mapping studies of humans with essential tremor has revealed a mutation in the Ten-m4 gene which correlates well with what has been observed in this model (Hor et al., 2015). Analysis of zebrafish morpholinos for Ten-m4 also showed changes in myelination as well as defects in motor axon pathfinding (Hor et al., 2015). The role of Ten-m4 in regulating the formation of visual or cortical circuits has yet to be reported.

## Concluding Remarks

These studies reviewed above demonstrate multiple, complex and important roles for teneurins in the formation and function of neural circuits. Their ability to mediate homophilic interactions is clearly crucial for the formation of precisely mapped connections between afferent and target fields in these circuits. Each of the teneurins, however, contains multiple domains and cleavage sites that may allow these molecules to also undergo heterophilic interactions with other key signaling molecules, such as the latrophilins (Boucard et al., 2014; Vysokov et al., 2016, 2018; Berns et al., 2018; Sando et al., 2019) as well as transcription factors such as Zic1 and Zic2 (Bagutti et al., 2003; Glendining et al., 2017). Thus, while expression patterns can help formulate hypotheses regarding function, other factors must also be taken into account when considering the roles of these highly complex molecules. Further information regarding the roles for different regions of these glycoproteins, the circumstances under which they are cleaved, and how this relates to their homophilic and heterophilic interactions is critical to a more comprehensive understanding of their function. The presence of multiple splice variants with differing binding properties is likely to add further to this complexity (Berns et al., 2018). The development of more refined tools such as conditional KOs will help to further reveal the manner in which this fascinating family of molecules interacts both at a circuit and cellular level to promote the proper wiring and function of critical sensory and learning networks. The demonstration that two members of the teneurin family play complimentary roles in enabling the generation of functional visual circuits is particularly intriguing, and together with evidence of conserved patterning across widely separated mammalian species tempts the speculation that they may have played crucial roles in the evolution of binocular vision in mammals.

## Author Contributions

Both authors contributed to the writing and editing of this manuscript.

## Conflict of Interest Statement

The authors declare that the research was conducted in the absence of any commercial or financial relationships that could be construed as a potential conflict of interest.
